# Perceptual discrimination of action formidableness and friendliness and the impact of autistic traits

**DOI:** 10.1038/s41598-024-76488-6

**Published:** 2024-10-26

**Authors:** Alessia M. Vlasceanu, Stephan de la Rosa, Nick E. Barraclough

**Affiliations:** 1 Department of Psychology, University of York, Heslington, York, YO10 5DD UK; 2 Department of Social Sciences, IU University of Applied Sciences, Juri-Gagarin-Ring 152, 99084 Erfurt, Germany

**Keywords:** Human behaviour, Sensory processing

## Abstract

The ability to determine whether the actions of other individuals are friendly or formidable are key decisions we need to make to successfully navigate our complex social environment. In this study we measured perceptual performance when discriminating actions that vary in their friendliness or formidableness, and whether performance was related to the autistic traits of individuals. To do this, we developed an action morphing method to generate novel actions that lied along the action quality dimensions of formidableness and friendliness. In Experiment 1 we show that actions that vary along the formidableness or friendliness continua were rated as varying monotonically along the respective quality. In Experiment 2 we measured the ability of individuals with different levels of autistic traits to discriminate action formidableness and friendliness using adaptive 2-AFC procedures. We found considerable variation in perceptual thresholds when discriminating action formidableness (~ 540% interindividual variation) or friendliness (~ 1100% interindividual variation). Importantly, we found no evidence that autistic traits influenced perceptual discrimination of these action qualities. These results confirm that sensory enhancements with autistic traits are limited to lower level stimuli, and suggest that the perceptual processing of these complex social signals are not affected by autistic traits.

## Introduction

Judgments of actions are critical to human social interactions. When observing the actions of another individuals, we derive multiple types of information from the action simultaneously in order to determine how to respond. The information available can range from simple kinematic information (e.g. action speed, fluency), to action goal (e.g. avoiding/approaching, lowering/raising), actor intentions (e.g. threatening, communicating) and actor traits (e.g. trustworthiness, dominance). This variety of information allows us to make efficient and appropriate behavioural and social responses to individuals acting within our complex social environment. Consequently, the ability to derive accurate information from the actions of other individuals is a key determinant of the success of our social interactions.

Although we can evaluate the actions of other individuals on a range of different characteristics, underlying these judgments are a smaller number of fundamental factors that account for a large proportion of the variance in the way we perceive actions. In a recent study^1^, we measured how 240 different motion-captured actions executed by a neutral avatar were perceived by observers. We identified 4 fundamental dimensions underlying visual action perception: formidableness (underlying judgments of action power, confidence, dominance etc.), friendliness (underlying judgments of action pro-sociality, happiness, trustworthiness etc.), planned (underlying judgments of action intentionality and control), and abduction (underlying judgments of whether the movement of limbs or objects are towards or away from the actor’s body). Each dimension extended from low negative values (e.g. feeble) to high positive values (e.g. formidable); whilst a value of zero indicated neither extreme (e.g. neither feeble nor formidable). These 4 action quality dimensions represent an ‘action space’, a low-dimensional solution that aims to model the conceptual framework in which all possible actions can exist (cf.^2–4^).

The ability to discriminate actions along these dimensions is important as they represent the fundamental qualities by which we make sense of other people’s actions. Whilst these action evaluations may also contribute to a more general framework of social cognition where key qualities of conspecifics are evaluated in terms of warmth and competence as a consequence of evolutionary pressures^5^. Evidence from the face domain supports this overall framework, as face traits appear to be evaluated on the fundamental dimensions of trustworthiness and dominance^6,7^, and could provide important adaptive information to the observer to infer behavioural intentions and social power hierarchies. Although the degree to which face trait information provides accurate information on which to guide behaviour remains contentious^8^. In contrast, when evaluating the ongoing dynamic actions of an individual, the ability to discriminate action friendliness provides important information on whether an actor intends to cause harm, whilst the ability to discriminate action formidableness provides information on whether an actor has the ability to do so. This information can be more informative than that available from static faces, particularly when face information is ambiguous^9^ or unavailable. Consequently, accuracy in action friendliness and formidableness discrimination may have important social benefits by providing fundamental social structural information (cf.^5^).

Importantly, there can also be considerable interindividual variation in the ability of individuals to discriminate visual information^10,11^, and discrimination performance is related to functional connectivity within the underlying neural systems involved in their processing^12^. However, we know little about action discrimination performance (although see:^13–15^), and it is currently unknown how the ability to discriminate the fundamental dimensions of friendliness and formidableness on which actions vary.

One important potential influence on the variability of individuals in the population to discriminate action qualities may be the degree to which they display autistic traits. Individuals with Autism Spectrum Condition (ASC) have difficulties in social perception^16,17^, including the evaluation of actions^18–23^, but see^24^. Due to the spectral nature of ASC, individuals without a diagnosis also display varying degrees of autistic traits. Individuals displaying higher (but not clinically significant) levels of autistic traits have been shown to display subtler versions of the behavioural and neurological characteristics associated with ASC (e.g.^15,25,26^). However, the evidence regarding deficits in social perception can be inconsistent with some reporting that individuals with ASC (e.g.^27–29^) or high levels of autistic traits (e.g.^30^) show typical performance on mentalizing tasks. Consequently, it remains currently unclear how autistic traits might impact the ability to accurately discriminate actions along the principal dimensions of action space of friendliness and formidableness. Assessing individual ability to discriminate these fundamental action qualities, that provide social structural information, allows us to understand their potential contributory role in the difficulties in social interactions observed in ASC^31^.

Potentially relevant to the perception of the action space dimension of friendliness, evidence points towards particular deficits in individuals with ASC when evaluating related social qualities. For example, children with ASC although able to understand the concept of individual trustworthiness, appear unable to use this information derived from facial features to consequently guide behaviour^32^. Similarly, adults with ASC were just as able as controls to recognise human actions conveyed by point-light stimuli, but were significantly poorer at recognising emotional point-light figures^33,34^. These deficits in action emotion perception in individuals with ASC compared to typically developing controls have been corroborated with both point-light body actions^35^, as well as photorealistic videos of actions^36^. However, in contrast to these findings, both behavioural measures, and blood-oxygen-level-dependent (BOLD) fMRI signals, to facial expressions of valence have been found to be similar between individuals with ASC and typically developing (TD) individuals (e.g.^37^). Despite this mixed picture, we tentatively predicted that autistic traits would have a detrimental impact on the discrimination of action friendliness.

Action formidableness refers to the power, speed, fluency, confidence and dominance conveyed by the action^1^. These action characteristics show considerable overlap with the traits that make up the dimension of ‘competence’ within the 2-dimensional model of social perception^5,38^; and judgments of competence underly positive social interactions^39^. Despite the potential importance of accurate judgments of action formidableness for social interactions, to our knowledge there is very little data on which to make any predictions about the likely influence of ASC on this ability. In one study, during the evaluation of non-verbal behaviour of virtual characters, individuals with high-functioning autism were no different in their evaluation of actor dominance compared with TD controls^40^. In a subsequent study Kuschefski, Falter‐Wagner^41^ also found no substantive differences between adults with ASC and TD controls when they rated the dominance and submissiveness of actors from their nonverbal interactions. Although some differences were observed in the speed at which the different individuals responded, with individuals with ASC being slower to respond. Together, this evidence suggests that autistic traits might not influence the ability to discriminate action formidableness.

To test these predictions, in this study we measured how well different individuals, who varied in their degree of autistic traits, could discriminate the friendliness and formidableness of different actions. First, we developed a novel action morphing technique in order to generate sets of actions that varied either on friendliness or formidableness, while controlling for the other quality. This method is particularly important, as these action qualities are naturally confounded, making it impossible to determine whether any perceptual deficits are due to the inability to discriminate action friendliness or action formidableness. Second, we tested how participants rated the degree of friendliness and formidableness of our novel morphed actions that varied along the dimensions of friendliness and formidableness. We expected that participants would only see the actions varying along the dimension varied. For example, actions that varied along the friendliness dimension should be seen as conveying varying amounts of friendliness, i.e. from unfriendly to friendly; whilst they would not be seen to convey different amounts of formidableness. Finally, we measured individual ability to discriminate action friendliness and action formidableness, and assessed interindividual variability in discrimination performance and whether this was related to the autistic traits of the participants measured with the AQ scale ^42^. Based upon the limited literature available, we predicted that action friendliness discrimination should be impaired by increasing autistic traits, whilst action formidableness discrimination would not be affected.

## Experiment 1: rating action formidableness and friendliness

### Methods

#### Participants

A total of 80 participants (72 females, 3 males, 5 other/prefer not to say, mean age = 18.86, SD = 0.84) took part in the study. A power analysis (using G*Power 3.1^43^) indicated that to detect a medium sized effect with a 2 × 6 factorial ANOVA 13 participants would be required, thus our sample of participants was suitably powered to detect differences in ratings of actions. All participants had normal or corrected to normal vision, were naive to the purpose of the study and provided written informed consent prior to the experiment. Participants received either course credits or were paid for taking part in the experiment. The study was approved by the Ethics Committee of the Department of Psychology, University of York, and was performed in accordance with the ethical standards laid down in the 1964 Declaration of Helsinki.

#### Stimuli

Action stimuli were generated from a set of 240 different motion capture actions available from the Open Science Framework at https://osf.io/4vew8/ that were used to examine action space^1^. In this prior study the loading of each of the 240 tested actions onto the 4 fundamental dimensions (friendliness, formidableness, planned, abducting) was calculated using Exploratory Factor Analysis (EFA). These loadings are equivalent to coordinates of each of the actions within the 4-dimensional action space. The values of these coordinates indicate how well a particular action conveys the 4 different action qualities. For example, one particular dancing action may have high (positive) values for formidableness, friendliness and abducting qualities, but a low (negative) value for the planned quality. Another dancing action may have different coordinates in action space, for example with average (zero) formidableness and abducting qualities, and high (positive) friendliness and planned qualities. Although we know the precise coordinates of the 240 actions in the dataset within action space, other actions not within the dataset in principal can exist at other coordinates.

We aimed to generate morphed actions that varied along one, but not the other dimensions. The first step in this process was to generate morphed action ‘prototypes’—actions that convey either a high (e.g. formidable) or low (e.g. feeble) amount of a single action quality, but no amount of other action qualities (e.g. friendliness). However, although we know the precise coordinates of each of the 240 actions in the dataset, the sample of 240 actions was not uniformly distributed across the 4-dimensional space. Because of this uneven distribution of source actions, it proved impossible to generate working prototype actions at either end of the 4 action quality dimensions. We instead focused on making prototypes for the dimensions of formidableness and friendliness, the two most important action space dimensions that explain the most amount of variance (together 44%) in action evaluation^1^ and ignored the degree to which the prototypes varied on the planned and abducting dimensions.

**Fig. 1:**
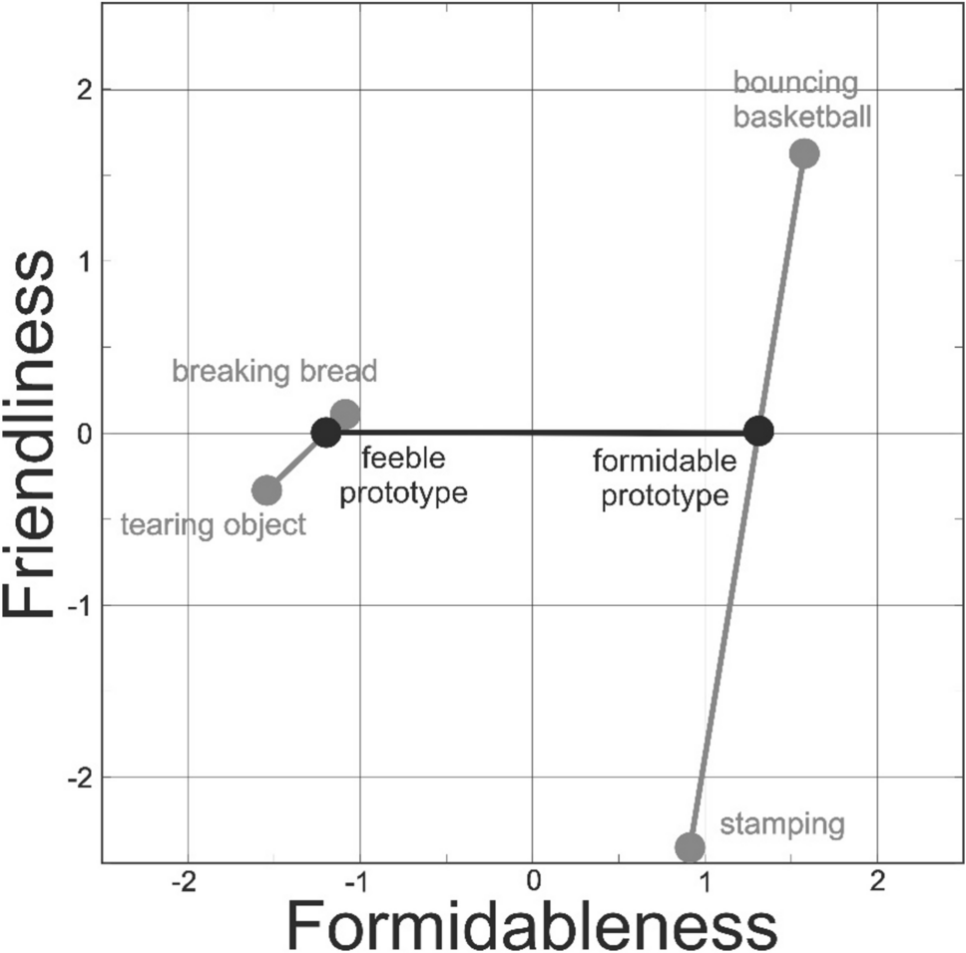
*Morphing process to generate actions along the formidable-feeble continuum.* *Source*: actions (grey markers) each have coordinates in the 4 quadrants of a 2D space: bouncing basketball (1.56, 1.62), stamping (.90, − 2.08), breaking bread (− 1.14, .13), tearing object (− 1.55, − .36). Weighted averages along the continuum between source actions (grey lines) are calculated to place feeble (− 1.25, 0) and formidable (1.27, 0) prototypes (black markers) on the feeble-formidable continuum. Stimuli used during the experiment are weighted averages along the formidable-feeble continuum between the prototypes (black line).

To generate the formidable and feeble prototypes, we selected one action from each of the 4 quadrants of a 2-dimensional version of action space (i.e. collapsed across dimensions 3 and 4) that showed similar durations and similar dominant moving body parts. Each action was processed so that they all lasted the same duration as the shortest action in the group (100 frames, 1.66s). One action was high in both formidableness and friendliness (bouncing a basketball), one action high in formidableness and low in friendliness (stamping on the ground), one action low in formidableness and high in friendliness (breaking a piece of bread), and one action low in both formidableness and friendliness (tearing an object into pieces). We then morphed between the two actions high in formidableness to generate the formidable prototype (see Fig. 1) by calculating the weighted average of the local joint angles of the bouncing a basketball action and the stamping on the ground action (using the same procedure as in^44–46^). The weights of the contributing actions were such as to ensure that the degree of friendliness conveyed by the resulting morph was zero. We used the same process to generate the feeble prototype by morphing between the breaking a piece of bread action and the tearing an object into pieces action. The formidable and feeble prototypes thus lay at high and low points on the formidableness continuum, whilst both actions lay at zero on the friendliness continuum. Finally, we then generated morphed actions along the continuum between the formidable and feeble prototypes by weighting the relative contributions of the prototypes in 1% steps from 100% formidable + 0% feeble through to 0% formidable + 100% feeble (see Fig. 1).

To generate actions that varied along the friendliness continuum we used a similar method as above but morphed between a different set of source actions. One action was high in both formidableness and friendliness (skipping; 2.46, 1.85), one action high in formidableness and low in friendliness (slamming hands down onto a table; − 2.05, 1.49), one action low in formidableness and high in friendliness (drinking; 1.49, − 0.41), and one action low in both formidableness and friendliness (crying; − 1.69, − 1.24). We morphed between the two actions high in friendliness (skipping, drinking) to generate the friendly prototype, and the morphed between the slamming hands down on the table and crying actions to generate the unfriendly prototype. Finally, we morphed (in 1% steps) between the friendly and unfriendly prototypes to generate morphed actions along the friendliness continuum.

**Fig. 2:**
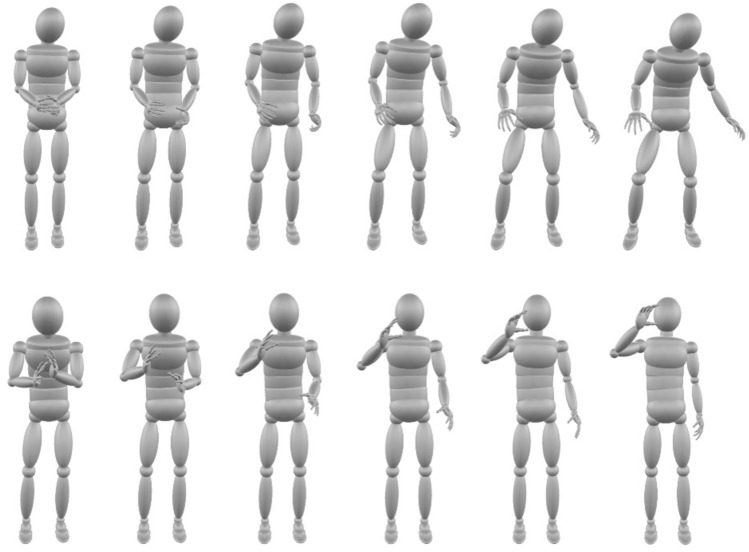
Action f*ormidableness and friendliness continua*. Illustrated are example frames taken 1 s into each video that contained (0%, 20%, 40%, 60%, 80%, 100%) of the formidable prototype (upper) or friendliness prototype (lower). The action showing 0% formidableness appears as a combination of breaking and tearing, the action showing 100% formidableness appears as a combination of bouncing and stamping. The action showing 0% friendliness appears as a combination of slamming and crying, the action showing 100% friendliness appears as a combination of skipping and drinking.

Morphing was conducted within the Unity 3D (Unity, San Francisco, CA. USA, https://unity.com) game engine by averaging between the joint angles recorded in the source action .bvh files. The resulting morphs were presented on screen by animating an androgynous volumetric avatar (see Fig. 2) where face, colour, texture, clothing, or identity information was not present. The only information available about the actions was the posture and motion of the avatar. To generate action videos for the experiment, Unity 3D played back each of the action morphs in 1% steps between the 2 prototypes on screen (1280 × 1080 pixels, 60fps), and playback was recorded with OBS Studio^47^ and each action was saved as an .mp4 file. For Experiment 1, actions along the formidableness continuum were selected that contained 0%, 20%, 40%, 60%, 80% and 100% of the formidable prototype, and actions along the friendliness continuum were selected that contained 0%, 20%, 40%, 60%, 80% and 100% of the friendly prototype.

#### Procedure

**Fig. 3:**
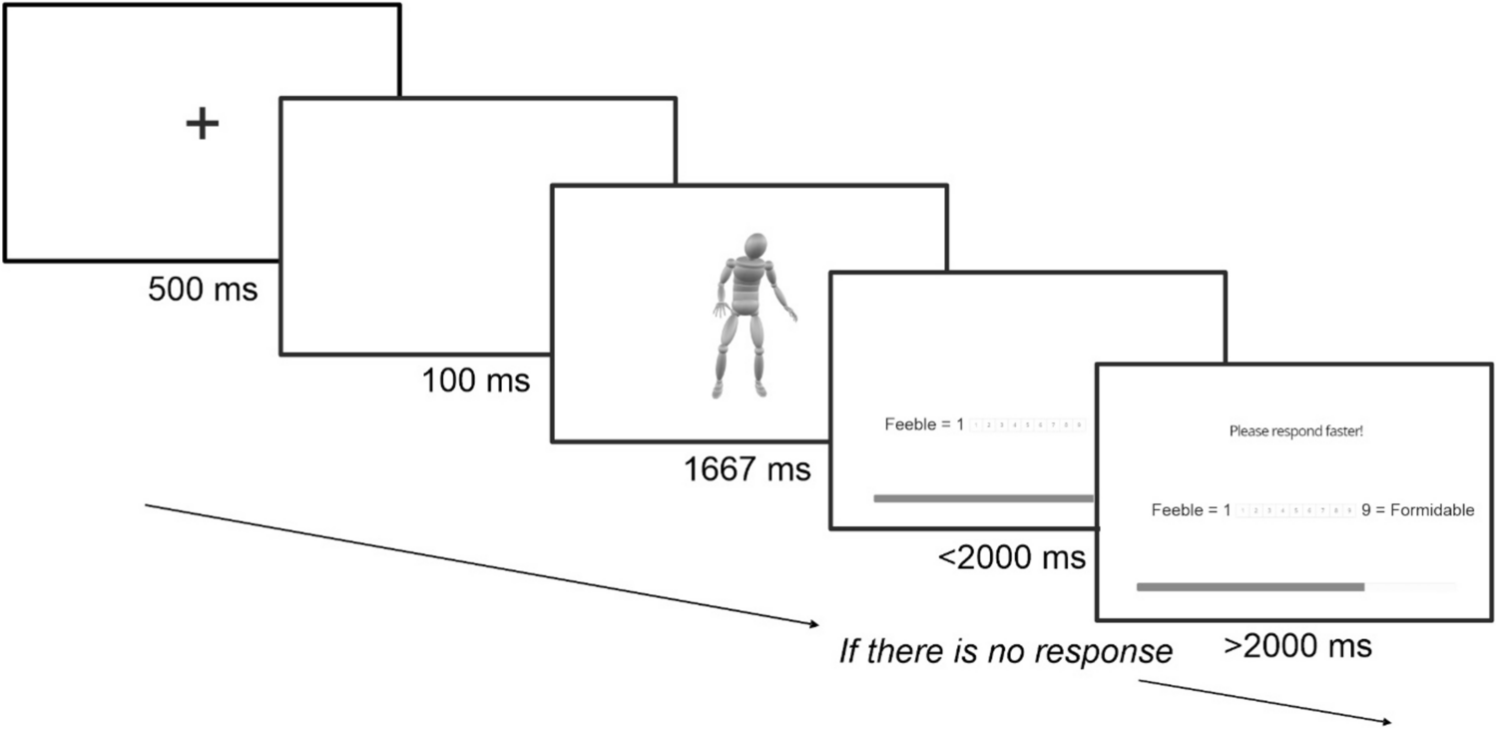
*Standard trial structure for the rating of action qualities.* Illustrated is a trial for the “Feeble—Formidable” quality. Following presentation of the action, response buttons are presented on screen, along with an experimental progress bar.

The experiment was implemented via the Gorilla Experiment Builder^48,49^. Once participants entered the experiment site through an internet browser on either a laptop or desktop computer, instructions on the experimental task were displayed and participants completed an informed consent form and entered simple demographic information (age and gender). Initially, participants took part in a set of 8 practice trials similar to those used during the experiment. On each trial (see Fig. 3), participants first viewed a 500 ms black fixation cross on a white screen, a 100 ms blank (white) screen, then the video of the action for its duration, and finally a 1–9 Likert scale (1 = unfriendly to 9 = friendly) where the participant had to indicate their immediate evaluation of the friendliness of the action by clicking an onscreen button with the mouse. If participants failed to respond within 2 s of the end of the action, a prompt “Please respond faster” appeared at the top of the response screen to encourage quick first impressions of the actions. Following completion of the practice trials, participants began the experiment itself.

The experiment consisted of 6 blocks of testing. Two blocks consisted of 12 trials where each of the 12 different actions were shown. In these two blocks participants were required to indicate either the formidableness (1 = Feeble to 9 = Formidable) or friendliness (1 = Unfriendly to 9 = Friendly) of the actions presented. Before each block, a set of task instructions were provided to the participants explaining the meaning of the quality (e.g. Formidableness) on which they were to evaluate the actions. During testing a progress bar was presented on screen along with the response buttons to provide participants with an indication of how far through the block they were. Block order was counterbalanced across participants. The other 4 blocks of testing were part of another experiment, the results of which are not reported here.

#### Analysis

For the action stimuli that varied along the formidableness continua, the aims were first to test whether there was an interaction between ratings of formidableness and friendliness (task) as action formidableness varied (morph level). Any interaction would indicate that perception of formidableness and friendliness varied differently when the level of action formidableness varied within the stimuli. Second, we assessed separately how ratings of formidableness or friendliness varied with morph level (equivalent to an analysis of the simple main effects), in order to understand how perception of these two action qualities varied with different levels of formidableness. We performed the equivalent analysis on the data where we varied the level of friendliness of the action stimuli. Analyses were conducted using repeated measures Bayesian ANOVAs implemented via JASP^50^, with uniform model priors.

### Results

**Fig. 4:**
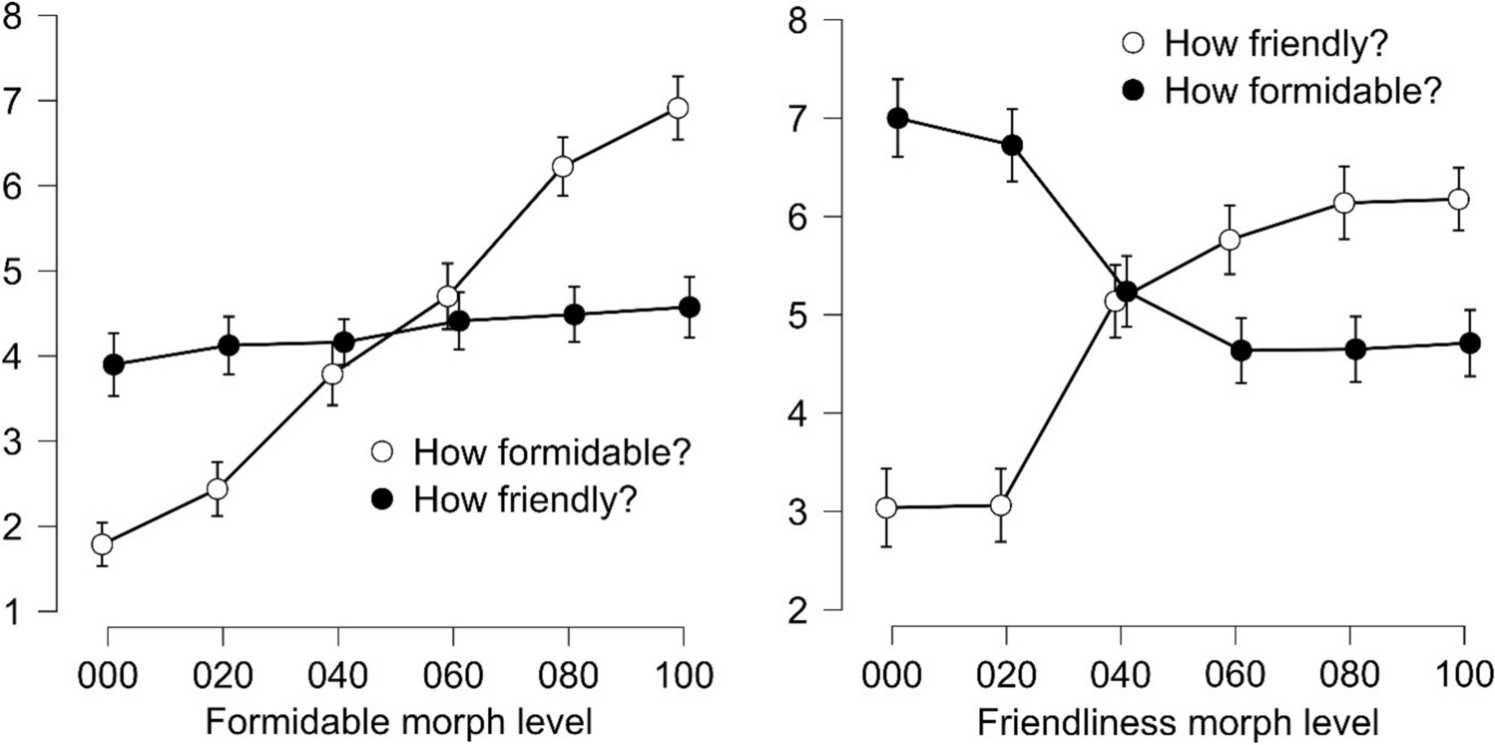
*Average rating of morphed actions along the formidableness and friendliness continua.* Left panel: actions varying along the formidableness continuum evaluated (on a 1–9 scale) on how formidable (open markers) and how friendly (closed markers) they appear. Right panel: actions varying along the friendliness continuum evaluated (on a 1–9 scale) on how friendly (open markers) and how formidable (closed markers) they appear. Error bars indicate 95% credible intervals.

We first compared the alternative hypothesis (H_1_) that there is an interaction between task and formidableness morph level with a null model (H_0_) that included the main effects of task and formidableness morph level. Following observation of the data, there was extreme evidence (BF_10_ = 9.51 × 10^49^, error % = 2.99) of an interaction between task and formidableness morph level. We then compared the alternative hypothesis that perception of formidableness varies with formidableness morph level with the null model. Following observation of the data, there was extreme evidence (BF_10_ = 2.17 × 10^99^, error % = 1.05) of perception of formidableness varying with formidableness morph level. Finally, we compared the alternative hypothesis that perception of friendliness varies with formidableness morph level with the null model. Following observation of the data, there was anecdotal evidence (BF_10_ = 0.87, error % = 0.48) for the null hypothesis that perception of friendliness does not vary with formidableness morph level. Thus, as the level of formidableness of the morphed action stimuli increases, perception of the level of formidableness increases monotonically, whilst it is unclear how this impacts the perception of the level of friendliness (see Fig. 4 left panel). These results show that actions that are morphed along the formidableness dimension are perceived as varying along this quality.

Our aims for the analysis or the action stimuli that varied along the friendliness continua were comparable to those above for the stimuli that varied along the formidableness continua, and thus we analysed the results in an equivalent fashion. We first compared the alternative hypothesis (H_1_) that there is an interaction between task and friendliness morph level with a null model (H_0_) that included the main effects of task and friendliness morph level. Following observation of the data, there was extreme evidence (BF_10_ = 2.19 × 10^85^, error % = 2.26) of an interaction between task and friendliness morph level. We then compared the alternative hypothesis that perception of friendliness varies with friendliness morph level with the null model. Following observation of the data, there was extreme evidence (BF_10_ = 3.25 × 10^55^, error % = 0.43) of perception of friendliness varying with friendliness morph level. Finally, we compared the alternative hypothesis that perception of formidableness varies with friendliness morph level with the null model. Following observation of the data, there was extreme evidence (BF_10_ = 1.02 × 10^36^, error % = 0.43) of perception of formidableness varying with friendliness morph level. Thus, as the level of friendliness of the morphed action stimuli increases, perception of the level of friendliness increases monotonically, however, the perception of the level of formidableness also concurrently decreases (see Fig. 4 right panel).

## Experiment 2: discriminating action formidableness and friendliness

### Methods

#### Participants

A power analysis (using G*Power 3.1^43^) indicated that to detect a medium sized effect with a linear regression model 55 participants would be required. We therefore recruited 55 participants who could discriminate actions along both the formidableness and friendliness dimensions. This required the testing of 64 participants in total, as 9 participants were excluded from statistical analyses as they could not discriminate actions along either one or both dimensions. Participants were adults recruited from the student population at the University of York, and received either course credits or were paid for taking part in the experiment; no demographic information was recorded. All participants had normal or corrected to normal vision, were naive to the purpose of the study and provided written informed consent prior to the experiment. The study was approved by the Ethics Committee of the Department of Psychology, University of York, and was performed in accordance with the ethical standards laid down in the 1964 Declaration of Helsinki.

#### Stimuli

Stimuli consisted of actions that varied in 1% steps along the formidableness and friendliness dimensions as prepared for Experiment 1.

#### Procedure

A PC running MATLAB 2021 and the Psychophysics Toolbox was used to control the experiment, present the stimuli, and record participant responses. Participants sat in a dimly lit room approximately 0.6 m away from a 27-in. TFT monitor (ASUS VP28U, 3840 × 2160 pixels, 60-Hz refresh rate) on which all action stimuli were presented.

We measured action quality discrimination thresholds (just noticeable differences: JNDs) using a 2-AFC procedure when participants: discriminated the formidableness of actions that varied along the formidable dimension (formidableness-formidable); discriminated the friendliness of actions that varied along the formidable dimension (friendliness-formidable); discriminated the friendliness of actions that varied along the friendliness dimension (friendliness-friendly); discriminated the formidableness of actions that varied along the friendliness dimension (formidableness-friendly). We predicted that participants would be able to discriminate the quality of actions on the dimension on which they varied; for example, participants should be able to discriminate the formidableness of actions that varied along the formidableness dimension. In contrast, we expected that participants would either be unable to, or would show poor performance, when attempting to discriminate the opposite action quality to that on which they varied; for example, participants would not be able to discriminate the friendliness of actions that varied along the formidableness dimension.

JNDs were measured for the 4 different conditions in 4 separate blocks of testing; conditions were counterbalanced across participants, although with 55 participants it was not possible to fully counterbalance all position and order effects. During a block of testing, the task involved the comparison of 2 sequentially presented actions on a particular action quality. For example, for the condition where participants had to discriminate the formidableness of actions that varied on formidableness: one action was the ‘standard’ (an action morph showing 50% formidableness), and the second a ‘comparison’ action. The degree to which the comparison action morph conveyed either formidableness or feebleness was determined by 4 interleaved staircases.

On each trial, participants first viewed a white screen displaying the quality on which the participant had to judge the actions (e.g. “Which action is more formidable”), followed by a 250-ms interval during which a black fixation cross appeared in the centre of the screen. Following the interval, the standard and comparison actions were presented 160-ms apart, the order of which was randomised. Participants had to indicate with a key press which of the two actions, first (key 1) or second (key 2), conveyed the most of the action quality (for example, which action was most formidable). On every trial, participants had to indicate a response, and they would only progress to the next trial once a response was registered. Once a response was registered, there was a 500 ms interval before the next trial. Whilst the standard remained the same, the comparison action was determined using adaptative staircase rules. There were 4 interleaved staircases with the following reversal rules: 1 up 2 down, 2 up 1 down, 1 up 3 down, 3 up 1 down. We did not determine thresholds from the staircase endpoints; instead procedures were used to distribute trials at informative points along the psychometric function^51^, which was fitted using the data from all the trials. Staircase step sizes were 8% and each staircase quit after 8 reversals, and the maximum number of trials per staircase type was 20, typically resulting in ~ 75 trials per psychometric function.

Strong order effects can result from perceptual learning and its significant impact on performance^52^. To mitigate these effects, each participant repeated the block for each condition until their performance plateaued and they showed no improvement in their ability to discriminate actions. We determined this by fitting a psychometric function to the data obtained from each block (of staircases), and once the JND calculated from data in block n was less than 1.5 standard deviation from the JND calculated from data in block n − 1, then no more data was collected for that condition. The coefficients from the function with the smallest JND for each condition were retained for subsequent analyses.

At the end of the experimental testing, participants had to complete an online version of the AQ questionnaire^42^. The AQ is a self-report measure of autistic traits consisting of 50 different questions. The test contains 10 questions each testing 5 different areas: social skill, attention switching, attention to detail, communication, imagination. Each question answer was scored to produce a total between 0 and 50, where a higher score indicates higher degrees of autistic traits. We separately recorded the subscale scores for these 5 different areas, as well as collating all scores to produce the final ‘AQ score’. Although, this test alone cannot diagnose the presence of Autism, it allowed us to compare the degree of autistic traits with the ability to discriminate actions along the 2 quality dimensions.

#### Analysis

For each participant and condition, JNDs were computed by first fitting cumulative Gaussian psychometric functions to the data using a maximum likelihood method of fit in MATLAB, while allowing the central tendency (mu) and the standard deviation (sigma) to freely vary. We divided the resulting standard deviations by √2 to give an estimate of the standard deviation on a single interval (because we used a 2-IFC procedure;^53^). The resulting values are JNDs because they indicate the percentage change in the action morph that can be discriminated at the ~ 76% level. The JNDs provide a measure of the ‘performance’ of the participants when discriminating the action morphs on a particular quality. Low JNDs (resulting from steep psychometric functions) indicated high sensitivity to the action quality, whilst high JNDs (resulting from gradual psychometric functions) indicated poor sensitivity to that action quality. We tested the influence of AQ on action discrimination thresholds with Bayesian linear regressions to quantify the evidence in favour for or against the alternative hypotheses that participants’ autistic traits predict their ability to discriminate formidableness and friendliness from actions.

### Results

The AQ scores and the AQ subscale scores (corresponding to measures of: Social skill, Attention switching, Attention to detail, Communication and Imagination) for all 55 participants are described in Table 1. Figure S1 in the Supplementary Information describes the distribution of these scores.

**Table 1:** Participant AQ scores and scores on the AQ subscales.

	Mean	Std	Min	Max
AQ	21.82	10.37	6	48
Social skill	3.71	2.87	0	10
Attention switching	5.83	2.33	1	10
Attention to detail	5.27	2.59	0	10
Communication	3.98	3.29	0	10
Imagination	2.98	2.06	0	10

**Fig. 5:**
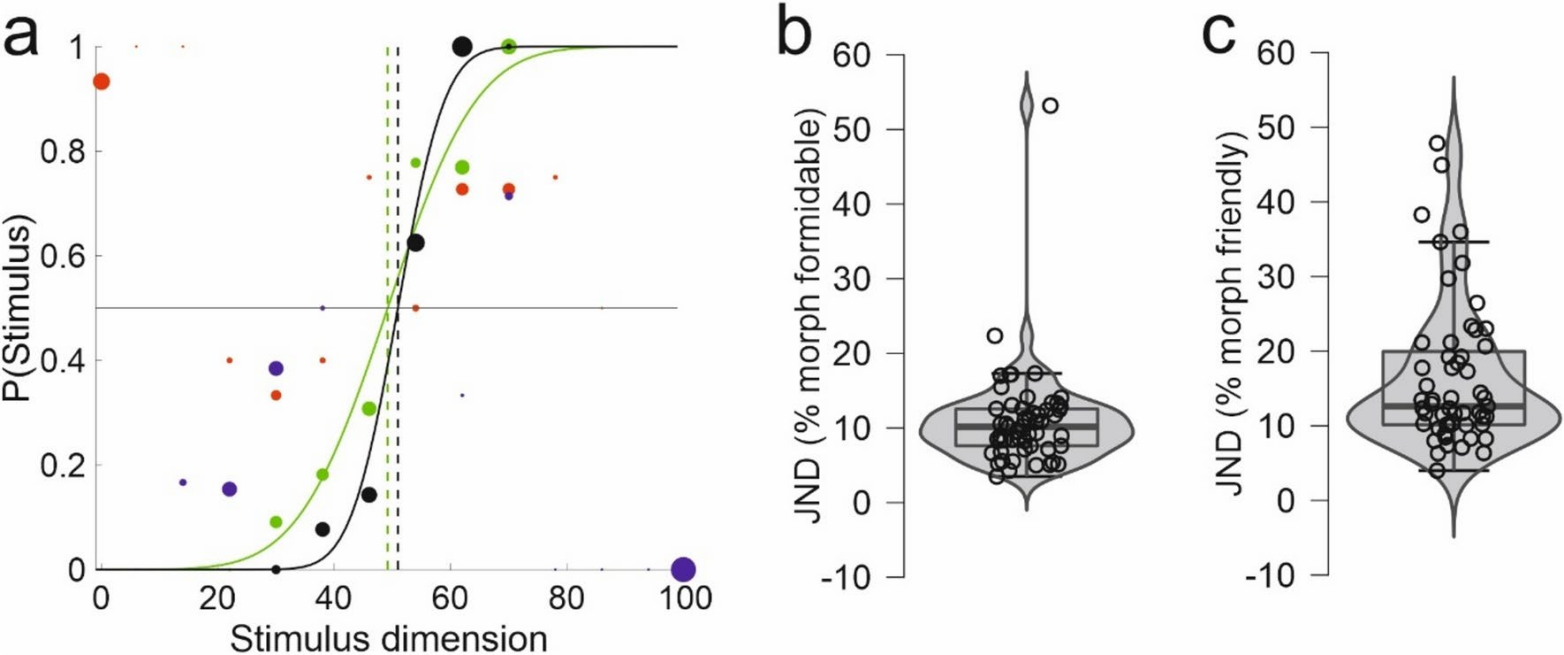
*Ability to discriminate action qualities from morphed actions.* (**a**) Psychometric functions fitted to the data from one individual for the friendliness-friendly condition (black), formidableness-formidable condition (green); the dotted lines indicate Mu the central tendency of each function. Markers illustrated data points, where circle magnitude corresponds to the number of trials for that data point. Psychometric functions could not be fitted to the data from the formidableness-friendly condition (red) or the data from the friendliness-formidable condition (blue). Cumulative Gaussian functions are fitted to the data points, the slopes of which are used to derive JNDs, for this example: discriminating friendliness of actions varying on friendliness (black, JND = 6.36%, SE = 1.26%), discriminating formidableness of actions varying on formidableness (green, JND = 12.02%, SE = 2.27%). (**b**) Violin and Box plots of all JNDs from the formidableness-formidable condition. (**c**) Violin and Box plots of all JNDs from the friendliness-friendly condition.

For all 55 participants we were able to fit psychometric functions to the data from both conditions where they discriminated the same action quality on which the actions varied. The mean JND for the condition where participants discriminated the formidableness of actions that varied along the formidable dimension (formidableness-formidable) was 11.00 (S.D. = 6.95), with considerable (~ 1400%) interindividual variation in JNDs (range 3.50 – 53.19). One individual showed a particularly large JND (53.19), however, even with this individual removed the interindividual variation in JNDs for discriminating formidableness was still substantial (~ 540%). The mean JND for the condition where participants discriminated the friendliness of actions that varied along the friendly dimension (friendliness-friendly) was 16.38 (S.D. = 9.78), again there was considerable (~ 1100%) interindividual variation in JNDs (range 4.00–47.85). For the condition where participants discriminated the formidableness of actions that varied along the friendliness dimension (formidableness-friendliness) we could not fit psychometric functions to the data for any participant, indicating that no one could discriminate the formidableness of actions that varied along the friendly continua. For the condition where participants discriminated the friendliness of actions that varied along the formidable dimension (friendliness-formidable) we could fit psychometric functions to the data for 17/55 (31%) of participants, indicating that a minority could still discriminate the friendliness of actions that varied along the formidable continua (Mean JND = 25.11, S.D. = 21.28) with considerable (~ 1100%) interindividual variation in JNDs (range 5.95–73.81). Figure 5 illustrates psychometric functions fitted to the data from an example individual, and the distribution of JNDs calculated from the psychometric functions fitted to the data for all participants.

**Fig. 6:**
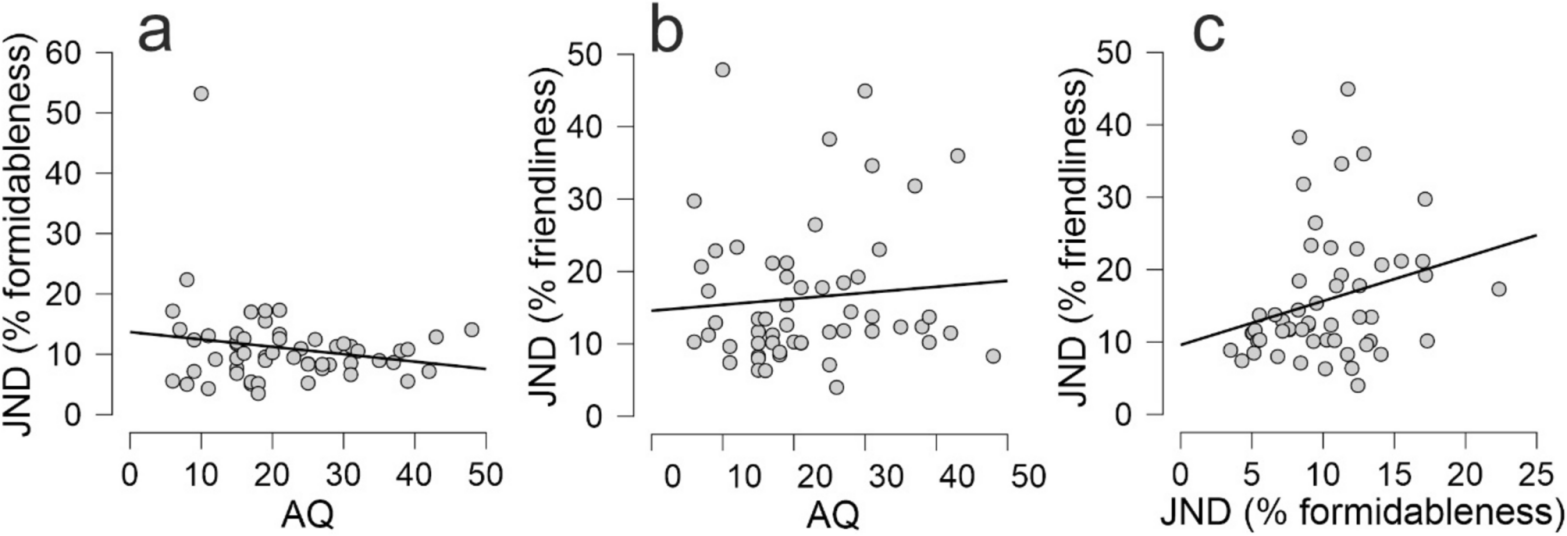
*Correlations with measures of action discrimination.* (**a**) Correlation between AQ score and JNDs when discriminating the formidableness of actions that varied along the formidable continua. (**b**) Correlation between AQ score and JNDs when discriminating the friendliness of actions that varied along the friendly continua. (**c**) Correlation between JNDs when discriminating the formidableness of actions that varied along the formidable continua and JNDs when discriminating the friendliness of actions that varied along the friendly continua, once one individual had been removed (N = 54).

There was not an obvious relationship between AQ and either discrimination of formidableness or friendliness (see Fig. 6a, b). Indeed, following observation of the data there was anecdotal evidence (BF_10_ = 0.40) in favour of the null hypothesis that AQ was not correlated (r = −0.183) with action formidableness discrimination, and moderate evidence (BF_10_ = 0.21) in favour of the null hypothesis that AQ was not correlated (r = 0.088) with action friendliness discrimination. We additionally correlated AQ subscale scores with discrimination performance for both formidableness and friendliness. Following observation of the data there was anecdotal evidence in favour of the null hypothesis that Social skill and Attention to detail was not correlated with action formidableness discrimination, and moderate evidence that Attention switching, Communication and Imagination was not correlated with action formidableness discrimination. Following observation of the data there was anecdotal evidence in favour of the null hypothesis that Communication was not correlated with action friendliness discrimination, and moderate evidence that Social skill, Attention switching, Attention to detail and Imagination was not correlated with action friendliness discrimination. Full correlation matrixes for AQ subscale analyses can be found in the Supplemental Information (Tables S2 & S3).

In order to examine how autistic traits predicted perceptual performance when discriminating action formidableness and friendliness we calculated 2 separate (one for each dimension) Bayesian linear regression models, each with uniform model priors, to examine the predictive value of AQs on JNDs. Following observation of the data, the odds in favour of models where AQ predicted discrimination performance decreased. The Bayes factor (BF_10_ = 0.58, R^2^ = 0.033) indicated anecdotal evidence in favour of the null hypothesis that AQ does not predict discrimination performance when evaluating action formidableness. The Bayes factor (BF_10_ = 0.32, R^2^ = 0.008) indicated moderate evidence in favour of the null hypothesis that AQ does not predict discrimination performance when evaluating action friendliness. We additionally performed Bayesian multiple regressions with AQ subscale scores as predictors separately for both formidableness and friendliness discrimination performance. Similarly to the analysis with the full AQ scale, following observation of the data, the odds in favour of all combinations of AQ subscale models predicting discrimination performance decreased (all BF_10_ < 1; see Tables S4 & S5 in the Supplementary Information for a full description of all model predictions).

Finally, to evaluate how perceptual performance on one action discrimination task was related to perceptual performance on the other action discrimination task we performed a Bayesian correlation and Bayesian linear regression between formidableness JNDs and friendliness JNDs. Following observation of the data there was extreme evidence (BF_10_ = 265) that action formidableness discrimination was correlated (r = 0.500) with action friendliness discrimination. The odds in favour of a model where action formidableness discrimination performance predicted action friendliness discrimination performance increased. The Bayes factor for the linear regression (BF_10_ = 220, R^2^ = 0.25) indicated extreme evidence in favour of formidableness discrimination performance predicting friendliness discrimination performance. However, the single individual with a particularly large JND for formidableness discrimination had a particularly large impact on evaluating the relationship between the two sets of JNDs. Once this individual was removed from the analysis following observation of the data there was only anecdotal evidence (BF_10_ = 1.07) that action formidableness discrimination performance was correlated with action friendliness discrimination performance (r = 0.277, see Fig. 6c). The odds in favour of a model where action formidableness discrimination predicted action friendliness discrimination increased. Whilst the Bayes factor (BF_10_ = 1.37, R^2^ = 0.071) indicated anecdotal evidence in favour of formidableness discrimination performance predicting friendliness discrimination performance.

## General discussion

The results of Experiment 1 showed that the morphing process resulted in two sets of actions that were seen as varying along either the formidableness or friendliness dimensions of action space respectively. For the formidable actions, as the level of formidableness of the actions increased, perception of the level of formidableness increased monotonically, whilst there was inconclusive evidence of the perception of friendliness varying. This showed that the actions morphed precisely along the formidableness dimension were perceived as varying along this quality only. For the friendly actions, as the level of friendliness of the actions increased, perception of the level of friendliness increased monotonically, however, there was also evidence of the perception of formidableness decreasing monotonically. This showed that the actions morphed precisely along the friendliness dimension did not isolate friendliness as originally intended.

The results of Experiment 2 showed that there was substantial interindividual variation in the ability to discriminate action formidableness and friendliness. The ability to discriminate formidableness varied by about 540% and friendliness varied by about 1100%. In comparison with previous measures of perceptual discrimination thresholds (e.g.^10^) this represents greater variability. Halpern et al.’s (1999) measures of perceptual discrimination were derived from lower level visual proficiency tests (e.g. measures of orientation discrimination, wavelength sensitivity, velocity discrimination, vernier acuity etc.). Here they typically observed variations in perceptual performance in the 60–100% range, although they also observed some larger variance (~ 1000%) in vernier acuity scores. For such lower level tests, there are likely to be fewer psychological and neural factors that impact performance. Even with such tests, variation in visual circuitry may explain interindividual discrimination variability, including foveal cone density, lateral geniculate nucleus (LGN), V1 volume, and V1 surface area. Attention is also likely to have an impact on these visual thresholds, as well as in the action discrimination tasks here. The ability to complete the discrimination tasks in our study will also rely on additional circuitry within other brain networks, including elements of the wider action observation network^54,55^, as well as networks involved in deriving emotional^56,57^ or dominance information^58^, that will likely bring further interindividual variation. The approximately two-fold difference in variability between formidableness discrimination thresholds and friendliness discrimination thresholds may reflect the relative complexity of the different tasks. Discrimination of action formidableness is derived from some assessments of action characteristics that could be achieved earlier in the visual processing hierarchy, including delineating action speed, fluency or power. In contrast, discrimination of action friendliness relies on the ability to assess action happiness, pro-sociality, approval, desire and trustworthiness^1^, all of which are comparatively more abstract concepts that are unlikely to be differentiated by simple visual processing mechanisms (although see:^59^). Furthermore, the paucity of evidence for a correlation between action formidableness discrimination performance and action friendliness discrimination performance shows that these tasks are performed by separate cognitive systems. In summary, our results suggest that measures of interindividual variance of perceptual discrimination performance of different stimulus qualities are related to the complexity of processing required to perform the task.

Our results provide evidence that discrimination of action formidableness and friendliness is not affected by the participants’ autistic traits. Indeed, following observation of the data the odds in favour of models where autistic traits predicted discrimination performance decreased. An important distinction between our analyses and the majority of previous studies that have examined the impact of ASC on the perception of formidableness (dominance) and friendliness (valence) is that we have employed Bayesian statistics. This allows the quantification of evidence of the null effect^60,61^: that autistic traits have no impact on the ability to discriminate action formidableness or friendliness. In addition, we used an adaptive discrimination task, which allows a direct measure of perceptual processing performance distinct from decision making processes, which are often confounded in behavioural tasks. Similar discrimination tasks to ours have often been used to measure the impact of ASC on sensory discrimination of relatively simple auditory, tactile and visual stimuli (e.g.^62–65^). And this prior research often demonstrates enhanced sensory discrimination in ASC which may explain some aspects of the ASC perceptual phenotype^66^. To our knowledge, however, ours is the first attempt to measure perceptual discrimination performance of such complex socially important qualities using these methods. Our results confirm that perceptual performance enhancements with autistic traits are restricted to ‘lower level’ stimuli.

Our results showed no evidence of autistic traits influencing the ability of individuals to discriminate formidableness from human actions. This appears to be in accordance with the findings of Schwartz, Dratsch^40^, and Kuschefski, Falter‐Wagner^41^, who found no effect of ASC diagnosis on ratings of dominance of interacting dyads. As in our experiment, these other two studies used computer avatars to eliminate other potential sources of social information from the face, scene, clothing etc. However, a difference was found in the speed of responses between ASC and TD individuals by Kuschefski, Falter‐Wagner^41^. They argued that this difference may have reflected TD individuals being able to complete the task using implicit reasoning and therefore they are quicker than individuals with ASC who require more effortful explicit reasoning despite equivalent success rates. Ours was an explicit task (direct instructions were given to participants), and measures the performance of the perceptual systems underlying the ability to derive formidableness information from the power, speed, confidence, dominance and fluency of the different actions. Consequently, the task is particularly affected by the ability to extract complex patterns of motion from the actions and so does not fully capture any impact of autistic traits on cognitive processing ‘styles’^67,68^ when discriminating action formidableness. Furthermore, during our task, participants were able to complete it at their own pace, responses were not timed nor recorded, and so currently it remains unclear how autistic traits may impact the time to it takes to discriminate the formidableness of another agent’s actions.

We also found no evidence of autistic traits influencing the ability of individuals to discriminate friendliness from human actions, indeed there was moderate evidence that autistic traits do not impact action friendliness discrimination. This corroborates previous studies showing that behavioural measures of the perception of emotions, at least in faces, can be similar between ASC and TD individuals^37,69,70^. However, recording of the BOLD response with fMRI (e.g.^69^) has shown that neural activity is different in ASC and TD individuals during emotion recognition tasks, suggesting the reliance on different cognitive processes to achieve comparable behavioural performance. In contrast to these findings, and our results, research examining recognition of emotions from point-light stimuli that may be more comparable to our actions, find opposite effects. Adults^33^, and children^34^, with ASC perform more poorly when labelling or naming emotions. Indeed, a study testing the ability to correctly label the emotion from photorealistic body language videos also showed deficits in children with ASC^71^.

So, why might our results agree with some data but not others? Although all our participants were adults, age related differences in emotional processing between children and adults cannot explain the differences. Adults with ASC have shown both deficits in emotion labelling (e.g.^33^) but also typical performance (e.g.^37^). Neither can the nature of the stimulus conveying the emotional information as studies using stimuli more similar to ours find deficits between ASC and TD individuals (e.g.^71^). We examined the impact of autistic traits, whereas the majority of studies examining emotion perception compared between groups of individuals with a clinical diagnosis of ASC and TD individuals. However, our approach does not preclude finding an impact of autistic traits on the perception of actions (e.g.^15^) in a population of health adults, whilst the range of AQ scores in our study (6–48) showed considerable variation in the levels of autistic traits in our sample population. However, the nature of our task is different from these others. In our study, we measured perceptual discrimination thresholds, and these reflect the performance limit of the underlying perceptual systems required to delineate the friendliness of similar actions from their movement and posture. Perhaps, other studies showing problems with emotional processing of actions may instead reflect co-occurring alexithymia^72^ or social anxiety^73^ often observed in ASD.

Our new action morphing method allows the generation of novel actions that can be varied precisely along any action quality of interest, whilst controlling for others. Typically, different action qualities are naturally confounded. For example, a walking actor may look purposeful, friendly, dominant and enthusiastic all at the same time. When using naturalistic actions—actors in photorealistic videos—it is not currently possible to vary the specific action qualities, nor attempt to control for other qualities. Our method, however, can help generate actions that allow the testing of the perception of specific action qualities. It also allows the generation of actions that vary in fractional steps along a continuum, therefore allowing discrimination performance tasks in the laboratory that cannot be conducted with photorealistic videos.

We used actions that were selected from the database of actions (https://osf.io/4vew8/) that are located within Vinton and colleagues’ 4-dimensional action space^1^. We isolated action friendliness and formidableness here, but the other dimensions of intentionality and abduction could also be isolated in the same fashion. Furthermore, in principle, it should be possible to generate novel actions that vary on one of these qualities while controlling for all the other three dimensions. This would require the morphing between 16 actions using the same methodological principles used here when morphing between 4 actions. Although this would require a wider availability of source actions than was available to us from the current dataset. Indeed, our method could be used with different source actions located with alternate action spaces (e.g.^74–77^). Although this would require the use of motion capture of different actions as these studies used photorealistic actions. But novel morphed actions could be made so as to vary along alternate dimensions identified as being important for social decision making (e.g. degree of sociality, see^77^).

However, there are some limitations to the methods we developed. For the actions morphed along the dimension of friendliness, we failed to control formidableness so that it did not vary at all. Instead the level of formidableness decreased as friendliness increased. Although action space dimensions are distinct, we cannot presume they are completely unrelated. Most psychological factors are correlated to some extent (discussed in Fabrigar, Wegener et al. 1999, Schmitt 2011), and this applies to the structure of the 4-dimensional action space^1^. This is also reflected in that the 4 dimensions were best modelled with an oblique factor structure allowing for likely correlations between dimensions. Even if action space was modelled with an orthogonal factor structure, and there was zero correlation between dimensions, this is still not evidence that the dimensions are statistically independent^78^. As such, varying friendliness has inadvertently allowed variation on formidableness. The selection of different source actions to generate morphed friendly actions may improve the isolation of that quality. Finally, it has yet to be determined how the generation of intermediary morphed actions not only influences the perception of the quality dimension of interest, but changes the perception of the action goal. The recognition of action goals is likely to have some impact on how more superordinate action qualities are perceived. Nether-the-less, our morphing method allows the generation of novel actions that convey increasing degrees of a specific quality whilst preventing a concurrent increase of other action qualities.

Finally, it is worth mentioning, that we did not expect that individuals would be able to discriminate actions that were morphed along one dimension, along the ‘opposite’ dimension. Indeed, no psychometric functions could be fitted to the data from any participant when they attempted to discriminate the formidableness of actions varying along the friendliness continua. Given that as action friendliness increased, ratings of formidableness decreased (see Fig. 4 right panel) attempts to discriminate formidableness from these stimuli during this task would have driven the staircases to diverge, rather than converge. This would have the effect of distributing the data sub-optimally, preventing the fit of psychometric functions. For a minority of individuals, functions could be fitted to the data when participants attempted to discriminate the friendliness of actions varying along the formidableness continua. Rating data for these stimuli (Fig. 4 left panel) indicates little variance in friendliness with formidable morphs, with only anecdotal evidence that there was no relationship. The mean of the JNDs calculated from this subset of individuals was substantially larger than the mean of the JNDs when participants discriminated formidableness of actions varying on formidableness, demonstrating the difficulty of the task.

In conclusion, we have developed a new action morphing method between multiple source actions that allows the generation of novel actions that vary, and are seen as varying, along fundamental dimensions on which actions can vary. These stimuli allowed us to measure perceptual discrimination thresholds of complex social signals across a range of individuals that varied in autistic traits. The ability to discriminate formidableness and friendliness from actions varied considerably between individuals, but this interindividual variation is not explained by levels of autistic traits in the individuals themselves. These findings confirm that sensory discrimination enhancements with autistic traits are limited to lower level stimuli, and suggest that variance in social perception with autistic traits are likely to be attributed to other causes, including variance in cognitive styles and social anxiety.

## Supplementary Information

Supplementary Information.

## Data Availability

We report how we determined our sample sizes, all data exclusions (if any), all manipulations, and all measures in the study, and we follow JARS^79^. Data collected during this study, their analysis using JASP, and movies of the morphed actions are all available publicly at: https://osf.io/qkw27.
