# Supplementary material for: Perceptual discrimination of action formidableness and friendliness and the impact of autistic traits
Source: Sci Rep. 2024 Oct 26;14:25554. doi: 10.1038/s41598-024-76488-6 (PMC11513001; doi:10.1038/s41598-024-76488-6)
Supplement: Supplementary file 1 — Supplementary Information. [file 41598_2024_76488_MOESM1_ESM.docx]

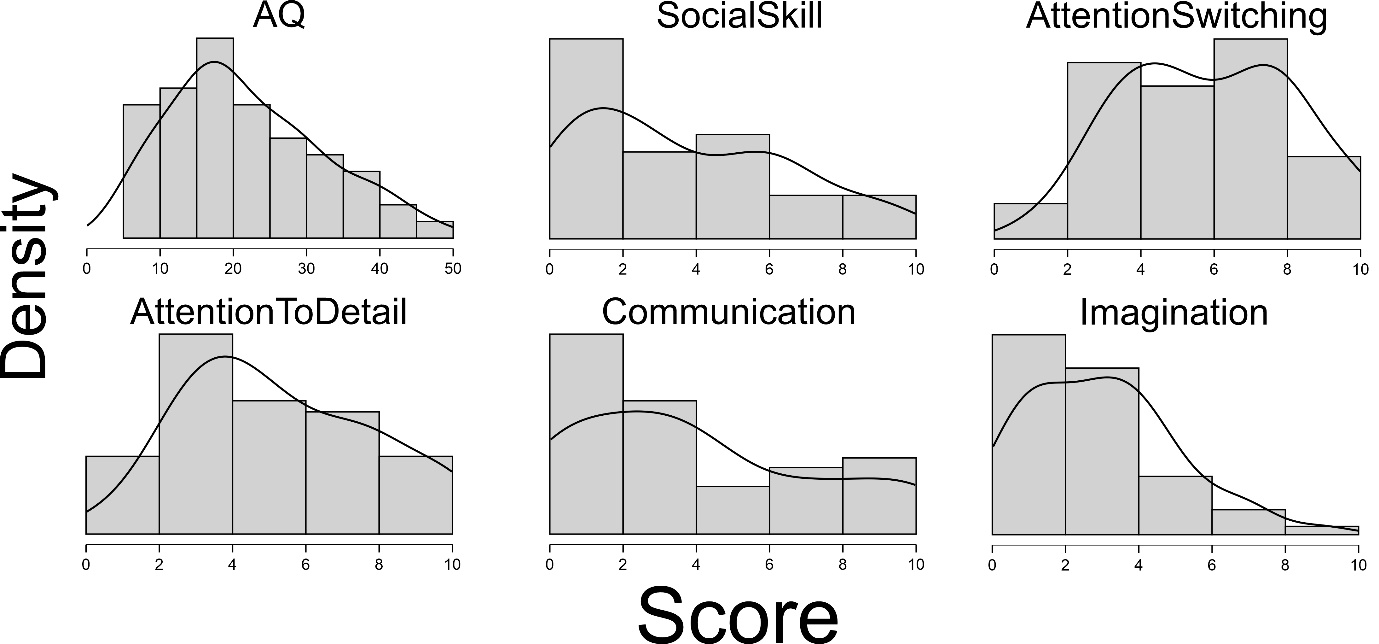


**Figure S1.** *Frequency distributions of scores derived from the AQ questionnaire*. Top left panel shows the distribution of AQ scores; other panels show the distributions of AQ subscales. The 5 subscale scores are summed to generate the final AQ score. The superimposed line on each plot indicates score density.

**Table S2**

*Bayesian Pearson Correlations between formidableness discrimination and AQ subscale scores*

|  |  | Formidableness | Social skill | Attention switching | Attention to detail | Communication |
| --- | --- | --- | --- | --- | --- | --- |
| Formidableness |  |  |  |  |  |  |
|  |  |  |  |  |  |  |
| Social skill | Pearson’s r | -.191 |  |  |  |  |
|  | B_10_ | .436 |  |  |  |  |
| Attention switching | Pearson’s r | -.113 | .696 |  |  |  |
|  | B_10_ | .234 | 3.54x10^+6^ |  |  |  |
| Attention to detail | Pearson’s r | -.174 | .395 | .395 |  |  |
|  | B_10_ | .369 | 12.82 | 12.750 |  |  |
| Communication | Pearson’s r | -.137 | .759 | .764 | .383 |  |
|  | B_10_ | .273 | 5.82x10^+8^ | 8.90x10^+8^ | 9.627 |  |
| Imagination | Pearson’s r | -.088 | .486 | .520 | .202 | .486 |
|  | B_10_ | .205 | 162.893 | 527.541 | .489 | 166.851 |

**Table S3**

*Bayesian Pearson Correlations between friendliness discrimination and AQ subscale scores*

|  |  | Friendliness | Social skill | Attention switching | Attention to detail | Communication |
| --- | --- | --- | --- | --- | --- | --- |
| Friendliness |  |  |  |  |  |  |
|  |  |  |  |  |  |  |
| Social skill | Pearson’s r | .047 |  |  |  |  |
|  | B_10_ | .178 |  |  |  |  |
| Attention switching | Pearson’s r | .067 | .695 |  |  |  |
|  | B_10_ | .189 | 3.54x10^+6^ |  |  |  |
| Attention to detail | Pearson’s r | .005 | .395 | .395 |  |  |
|  | B_10_ | .168 | 12.82 | 12.750 |  |  |
| Communication | Pearson’s r | .207 | .759 | .764 | .383 |  |
|  | B_10_ | .514 | 5.82x10^+8^ | 8.90x10^+8^ | 9.627 |  |
| Imagination | Pearson’s r | -.035 | .486 | .520 | .202 | .486 |
|  | B_10_ | .174 | 162.893 | 527.541 | .489 | 166.851 |

**Table S4**

*Bayesian Multiple Regression models for AQ subscale scores predicting formidableness discrimination performance*

| **Models** | **P(M)** | **P(M\|data)** | **P(M\|data)** | **BF10** | **R²** |
| --- | --- | --- | --- | --- | --- |
| Null model |  | .537 | 5.792 | 1 | 0 |
| SocialSkill | .033 | .67 | 2.076 | .622 | .037 |
| AttentionToDetail | .033 | .058 | 1.777 | .538 | .03 |
| Communication | .033 | .044 | 1.346 | .413 | .019 |
| AttentionSwitching | .033 | .039 | 1.171 | .362 | .013 |
| Imagination | .033 | .035 | 1.041 | .323 | .008 |
| SocialSkill + AttentionSwitching + AttentionToDetail + Communication + Imagination | .167 | .019 | .095 | .035 | .05 |
| SocialSkill + AttentionToDetail | .017 | .016 | .951 | .296 | .048 |
| SocialSkill + AttentionSwitching | .017 | .013 | .749 | .234 | .037 |
| SocialSkill + Communication | .017 | .012 | .74 | .231 | .037 |
| SocialSkill + Imagination | .017 | .012 | .737 | .23 | .037 |
| AttentionToDetail + Communication | .017 | .012 | .729 | .227 | .036 |
| AttentionToDetail + Imagination | .017 | .011 | .684 | .214 | .033 |
| AttentionSwitching + AttentionToDetail | .017 | .011 | .676 | .211 | .033 |
| Communication + Imagination | .017 | .009 | .508 | .159 | .019 |
| AttentionSwitching + Communication | .017 | .008 | .503 | .158 | .019 |
| AttentionSwitching + Imagination | .017 | .008 | .453 | .142 | .014 |
| SocialSkill + AttentionSwitching + AttentionToDetail | .017 | .007 | .431 | .135 | .05 |
| SocialSkill + AttentionSwitching + AttentionToDetail + Imagination | .033 | .007 | .207 | .066 | .05 |
| SocialSkill + AttentionSwitching + AttentionToDetail + Communication | .033 | .007 | .206 | .066 | .05 |
| SocialSkill + AttentionToDetail + Communication | .017 | .007 | .418 | .131 | .049 |
| SocialSkill + AttentionToDetail + Imagination | .017 | .007 | .411 | .129 | .048 |
| SocialSkill + AttentionToDetail + Communication + Imagination | .033 | .007 | .2 | .064 | .049 |
| SocialSkill + AttentionSwitching + Imagination | .017 | .006 | .329 | .103 | .037 |
| SocialSkill + AttentionSwitching + Communication | .017 | .006 | .329 | .103 | .037 |
| SocialSkill + Communication + Imagination | .017 | .005 | .325 | .102 | .037 |
| SocialSkill + AttentionSwitching + Communication + Imagination | .033 | .005 | .16 | .051 | .037 |
| AttentionToDetail + Communication + Imagination | .017 | .005 | .324 | .102 | .036 |
| AttentionSwitching + AttentionToDetail + Communication + Imagination | .033 | .005 | .158 | .051 | .037 |
| AttentionSwitching + AttentionToDetail + Communication | .017 | .005 | .321 | .101 | .036 |
| AttentionSwitching + AttentionToDetail + Imagination | .017 | .005 | .306 | .096 | .034 |
| AttentionSwitching + Communication + Imagination | .017 | .004 | .229 | .072 | .019 |

**Table S5**

*Bayesian Multiple Regression models for AQ subscale scores predicting friendliness discrimination performance*

| **Models** | **P(M)** | **P(M\|data)** | **P(M\|data)** | **BF10** | **R²** |
| --- | --- | --- | --- | --- | --- |
| Null model | .167 | .507 | 5.14 | 1 | 0 |
| Communication | .033 | .073 | 2.282 | .719 | .043 |
| SocialSkill + AttentionSwitching + AttentionToDetail + Communication + Imagination | .167 | .041 | .215 | .081 | .094 |
| AttentionSwitching | .033 | .03 | .91 | .3 | .004 |
| SocialSkill | .033 | .029 | .863 | .285 | .002 |
| Imagination | .033 | .028 | .845 | .279 | .001 |
| AttentionToDetail | .033 | .028 | .822 | .272 | 0 |
| SocialSkill + Communication | .017 | .025 | 1.539 | .501 | .071 |
| Communication + Imagination | .017 | .023 | 1.382 | .451 | .067 |
| AttentionSwitching + Communication | .017 | .021 | 1.251 | .409 | .063 |
| SocialSkill + AttentionSwitching + Communication + Imagination | .033 | .016 | .461 | .154 | .092 |
| AttentionToDetail + Communication | .017 | .015 | .92 | .303 | .049 |
| SocialSkill + Communication + Imagination | .017 | .015 | .897 | .295 | .087 |
| SocialSkill + AttentionToDetail + Communication + Imagination | .033 | .015 | .437 | .147 | .09 |
| SocialSkill + AttentionSwitching + Communication | .017 | .013 | .792 | .261 | .081 |
| SocialSkill + AttentionSwitching + AttentionToDetail + Communication | .033 | .013 | .378 | .127 | .083 |
| AttentionSwitching + AttentionToDetail + Communication + Imagination | .033 | .012 | .367 | .123 | .081 |
| AttentionSwitching + Communication + Imagination | .017 | .012 | .733 | .242 | .078 |
| SocialSkill + AttentionToDetail + Communication | .017 | .011 | .679 | .224 | .074 |
| AttentionToDetail + Communication + Imagination | .017 | .011 | .66 | .218 | .073 |
| AttentionSwitching + AttentionToDetail + Communication | .017 | .01 | .566 | .188 | .066 |
| AttentionSwitching + Imagination | .017 | .007 | .404 | .134 | .011 |
| SocialSkill + Imagination | .017 | .006 | .366 | .122 | .007 |
| AttentionSwitching + AttentionToDetail | .017 | .006 | .354 | .118 | .005 |
| SocialSkill + AttentionSwitching | .017 | .006 | .35 | .116 | .004 |
| SocialSkill + AttentionToDetail | .017 | .006 | .335 | .112 | .002 |
| AttentionToDetail + Imagination | .017 | .006 | .329 | .109 | .001 |
| SocialSkill + AttentionSwitching + AttentionToDetail + Imagination | .033 | .003 | .093 | .032 | .012 |
| AttentionSwitching + AttentionToDetail + Imagination | .017 | .003 | .186 | .062 | .012 |
| SocialSkill + AttentionSwitching + Imagination | .017 | .003 | .185 | .062 | .012 |
| SocialSkill + AttentionToDetail + Imagination | .017 | .003 | .168 | .056 | .007 |
| SocialSkill + AttentionSwitching + AttentionToDetail | .017 | .003 | .163 | .054 | .005 |
